# Rice nitrate transporter *OsNPF7.2* positively regulates tiller number and grain yield

**DOI:** 10.1186/s12284-018-0205-6

**Published:** 2018-02-27

**Authors:** Jie Wang, Kai Lu, Haipeng Nie, Qisen Zeng, Bowen Wu, Junjie Qian, Zhongming Fang

**Affiliations:** 1 Center of Applied Biotechnology, Wuhan Institute of Bioengineering, Wuhan, 430415 China; 2 National Key Laboratory of Crop Genetic Improvement, Huazhong Agricultural University, Wuhan, 430070 China

**Keywords:** Rice, *OsNPF7.2*, Tiller bud, Cytokinin, Tiller number, Grain yield

## Abstract

**Background:**

Rice tiller number is one of the most important factors that determine grain yield, while nitrogen is essential for the crop growth and development, especially for tiller formation. Genes involved in nitrogen use efficiency processes have been identified in the previous studies, however, only a small number of these genes have been found to improve grain yield by promoting tillering.

**Results:**

We constructed over-expression (OX) lines and RNA-interference (Ri) lines, and selected a mutant of *OsNPF7.2*, a low-affinity nitrate transporter. Our analyses showed that rice tiller number and grain yield were significantly increased in OX lines, whereas Ri lines and mutant *osnpf7.2* had fewer tiller number and lower grain yield. Under different nitrate concentrations, tiller buds grew faster in OX lines than in WT, but they grew slower in Ri lines and mutant *osnpf7.2*. These results indicated that altered expression of *OsNPF7.2* plays a significant role in the control of tiller bud growth and regulation of tillering. Elevated expression of *OsNPF7.2* also improved root length, root number, fresh weight, and dry weight. However, reduced expression of *OsNPF7.2* had the opposite result on these characters. *OsNPF7.2* OX lines showed more significantly enhanced influx of nitrate and had a higher nitrate concentration than WT. The levels of gene transcripts related to cytokinin pathway and cell cycle in tiller bud, and cytokinins concentration in tiller basal portion were higher in OX lines than that in WT, suggesting that altered expression of *OsNPF7.2* controlled tiller bud growth and root development by regulating cytokinins content and cell cycle in plant cells. Altered expression of *OsNPF7.2* also was responsible for the change in expression of the genes involved in strigolactone pathway, such as *D27*, *D17*, *D10*, *Os900*, *Os1400*, *D14*, *D3*, and *OsFC1*.

**Conclusion:**

Our results suggested that *OsNPF7.2* is a positive regulator of nitrate influx and concentration, and that it also regulates cell division in tiller bud and alters expression of genes involved in cytokinin and strigolactone pathways, resulting in the control over rice tiller number. Since elevated expression of *OsNPF7.2* is capable of improving rice grain yield, this gene might be applied to high-yield rice breeding.

**Electronic supplementary material:**

The online version of this article (10.1186/s12284-018-0205-6) contains supplementary material, which is available to authorized users.

## Background

Rice (*Oryza sativa* L.) is one of the three major grain crops grown worldwide and is consumed by more than half of the world’s population (Khush 2005). The rapid increase of the human population puts high demand on rice production, meanwhile high rice yield is a target pursued by plant breeders. Rice yield is mainly controlled by three factors: panicle number per plant, grain number per panicle, and thousand-grain weight. Panicle number per plant is dependent on the ability of plant to produce tillers (Liang et al. 2014). Starting with shoot branching, rice tiller experience two distinct stages in its development: the formation of an tiller bud at each leaf axil and the outgrowth of the tiller bud (Li et al. 2003; Xing and Zhang 2010). Therefore, final tiller number is determined not only by the number of tiller bud but also by outgrowth rate of tiller bud (Wang and Li 2011). In the past few years, many quantitative trait loci (QTLs) and genes involved in tiller bud formation and outgrowth in rice have been identified, such as *MOC1* (Li et al. 2003), *MOC2* (Koumoto et al. 2013), *MOC3*/*SRT1* (Lu et al. 2015; Mjomba et al. 2016), *TAD1*/*TE* (Xu et al. 2012; Lin et al. 2012), *LAX1* (Oikawa and Kyozuka 2009), *LAX2* (Tabuchi et al. 2011), *OsTB1*/*OsFC1* (Takeda et al. 2003; Minakuchi et al. 2010), especially, the genes responsible for strigolactone pathways, such as *D27* (Lin et al. 2009), *D17*/*OsCCD7*/*HTD1* (Zou et al. 2005; Zou et al. 2006; Kulkarni et al. 2014; Yang et al. 2017), *D10* (Arite et al. 2007), *D14* (Arite et al. 2009), *D3* (Ishikawa et al. 2005; Yoshida et al. 2012), and *D53* (Zhou et al. 2013; Jiang et al. 2013).

Tiller bud outgrowth is regulated not only by endogenous factors, but also by environmental signals (Xing and Zhang 2010). Nitrogen (N), as an important environmental factor, affects rice growth and development including rice tillering. Nitrate is the major form of N available in aerobic environments and many members of nitrate transporter gene families are found in rice, such as 80 NPFs (NRT1/PTRs: NRT1, low-affinity nitrate transporter; PTR, di/tripeptide transporter), 5 NRT2s, and 2 NAR2s members. To date, only a few NPF members have been characterized in rice (Li et al. 2017). *OsNRT1* (*OsNPF8.9*) was first described and found to function as a low affinity nitrate transporter (Lin et al. 2000). Afterwards, other *NPFs* were explored, such as *SP1* (*OsNPF4.1*) and *OsPTR9* (*OsNPF8.20*), however, their substrates remain unclear (Lin et al. 2000; Fang et al. 2013), Recently, *OsNPF2.4*, *OsNPF2.2*, and *OsNPF7.2* have been reported to serve as low-affinity nitrate transporters functioning under high nitrate concentrations (Li et al. 2015; Xia et al. 2015; Hu et al. 2016). Allelic differences in the dual-affinity nitrate transporter *NRT1.1B* (*OsNPF6.5*) have been reported between *indica* and *japonica* cultivars with high nitrogen-use efficiency and grain yield in the *NRT1.1B*–indica allele (Hu et al. 2015). OsPTR6 (OsNPF7.3) transports di/tripeptides Gly-His and Gly-His-Gly and its high levels of expression enhance rice growth (Fan et al. 2014). A recent study reveals that *OsNPF7.3* is induced by organic nitrogen, and that elevated expression of *OsNPF7.3* increases the number of panicles per plant, filled grain numbers per panicle, grain nitrogen content, and enhances grain yield (Fang et al. 2017). OsPTR7 (OsNPF8.1) shows dimethylarsenate (DMA) transport activity and is involved in the long-distance translocation of DMA into rice grain (Tang et al. 2017).

Of all the characterized NPF transporters to date, only *OsNPF8.20*, *OsNPF6.5*, and *OsNPF7.3* can moderate rice tiller number and enhance grain yield (Fang et al. 2013; Hu et al. 2015; Fang et al. 2017). It is unclear whether other NPF genes play a role in rice tillering, especially by regulating N and phytohormones in plant cells. One previous study showed that knock-out of *OsNPF7.2* retarded rice root growth under high nitrate supply (Hu et al. 2016). However, the effect of increased expression of *OsNPF7.2* on rice growth and development is yet unknown, neither is the influence agronomic traits. This study analysed over-expression lines (OX), RNA-interference lines (Ri), and a mutation of *OsNPF7.2* and found that over-expression of *OsNPF7.2* significantly increased rice tiller number by promoting tiller bud elongation and by regulating cytokinin (CK) and strigolactone (SL) pathway in cells.

## Results

### Over-expression of *OsNPF7.2* improves rice tiller number and grain yield

**Fig. 1:**
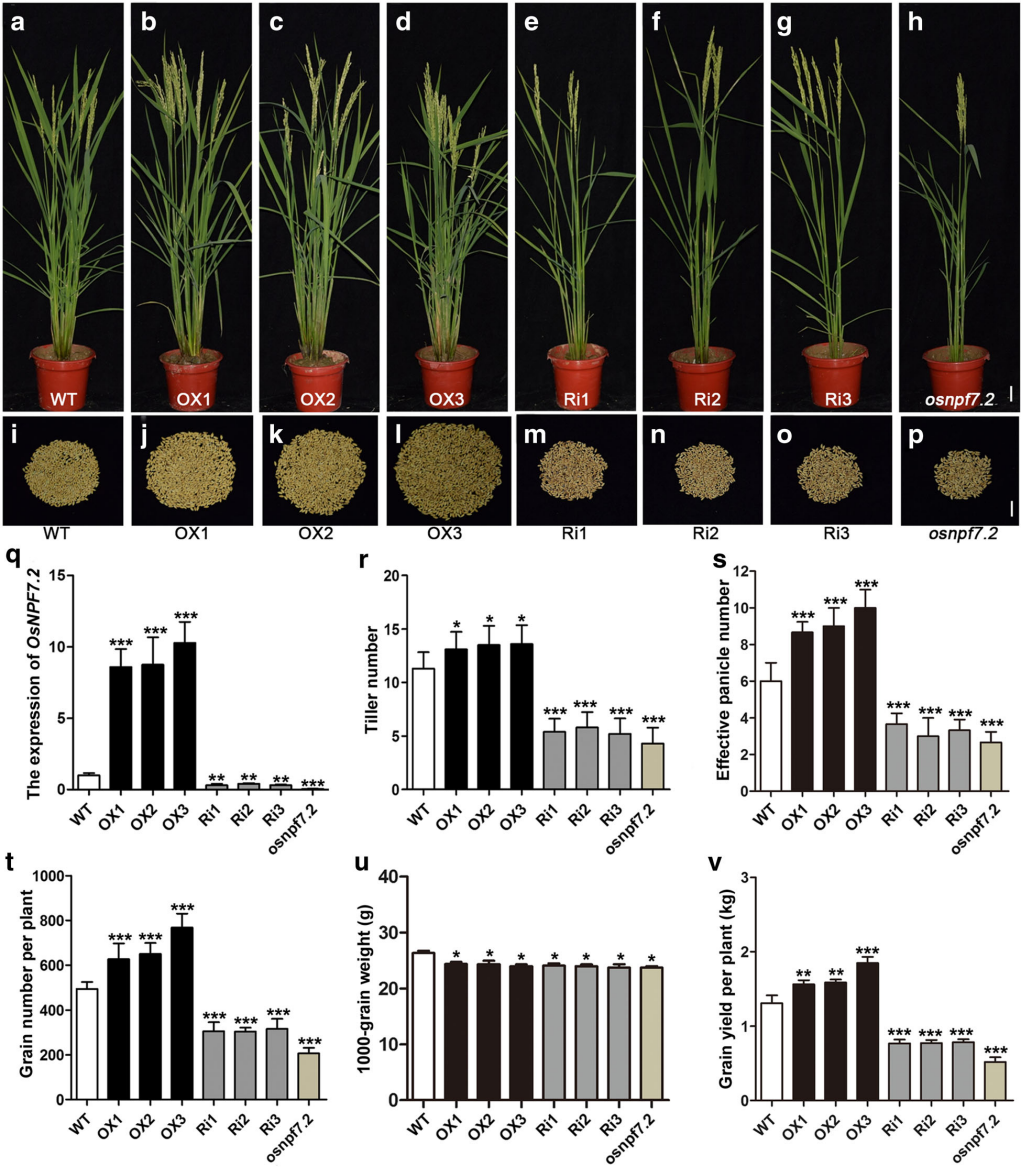
Characterization of transgenic lines of *OsNPF7.2*. **a-h** Phenotypic analysis of WT, OX lines, Ri lines and mutant *osnpf7.2* at mature stage. Bar = 5 cm. **i-p** Comparison of total grain number among WT, OX lines, Ri lines and *osnpf7.2*. Bar = 5 cm. **q** Transcript abundance of *OsNPF7.2* in tiller buds among WT, OX lines, Ri lines and *osnpf7.2*. **r-v** Comparison of agronomic traits including tiller number (**r**), effective panicle number (**s**), grain number per plant (**t**), 1000-grain weight (**u**) and grain yield per plant (**v**) among WT, OX lines, Ri lines and *osnpf7.2*. WT, OX and Ri were abbreviations for wild type, over-expression and RNA-interference, respectively. Data in **q-v** are shown as means ± SD (*n* = 10) from three replicates. A student’s t-test was used to generate *P* values; “*, ** and ***” indicate significance at *P* < 0.05, *P* < 0.01 and *P* < 0.001, respectively

*OsNPF7.2* is mainly expressed in the roots of seedlings, and its protein transports nitrate at vacuolar membrane (Hu et al. 2016). In order to investigate the effects of the altered expression of *OsNPF7.2* on rice growth and development, we constructed over-expression (OX) lines and RNA-interference (Ri) lines; we also analysed knock-out mutant *osnpf7.2*. We found that tiller number increased in three OX lines at reproductive stage (Fig. 1b-d, r) compared to that in wild-type (WT) ZH11 (Fig. 1a, r), but it dramatically decreased in three Ri lines (Fig. 1e-g, r) and mutant *osnpf7.2* (Fig. 1h, r). Three OX lines also had a higher total grain number per plant than WT (Fig. 1i-l, t), whereas Ri lines had a lower total grain number per plant than WT (Fig. 1m-o, t). The total grain number of mutant *osnpf7.2* was less than half of that of WT (Fig. 1p, t). It was confirmed that the formation of phenotype resulted from the altered expression of *OsNPF7.2* by using qRT-PCR in different transgenic lines (Fig. 1a-h and q). Overall, our results indicated that elevated *OsNPF7.2* expression level significantly enhanced the total grain number per plant.

The number of panicles derived from rice tillers is one of the three key factors determining rice grain yield (Xing and Zhang 2010). The analysis of panicle number in the transgenic lines presented similar change trend of tiller number as described above (Fig. 1s). Moreover, there was no significant difference in 1000-grain weights among different transgenic lines of *OsNPF7.2* (Fig. 1u). Grain yield per plant in OX lines was significantly greater than that in WT (Fig. 1v). Thus, over-expression of *OsNPF7.2* significantly increased rice tiller number and total grain number per plant, while down-regulation of *OsNPF7.2* produced the opposite effects.

### Elevated expression of *OsNPF7.2* promotes rice tiller bud outgrowth especially under high nitrate concentrations

**Fig. 2:**
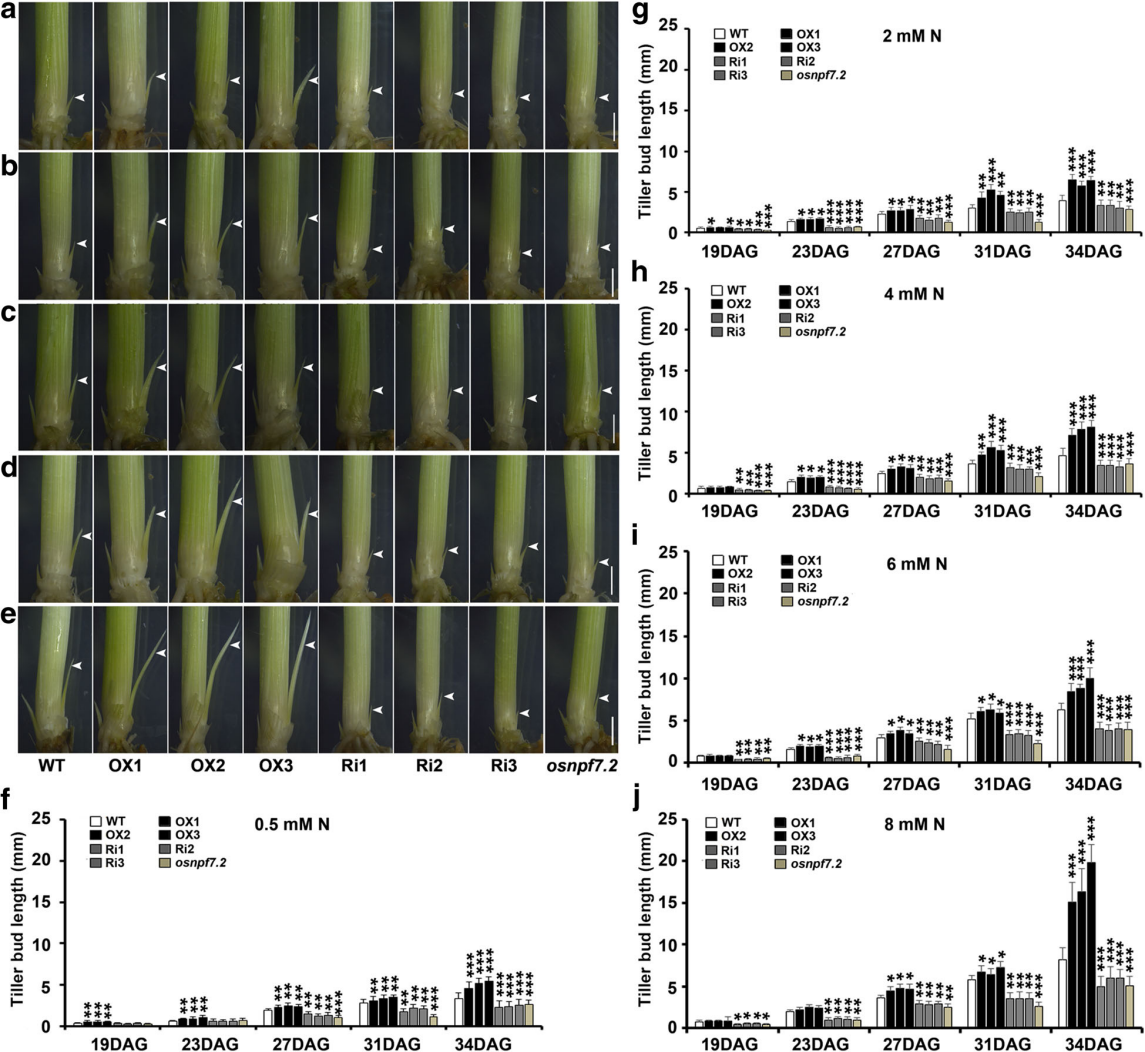
Altered expression of *OsNPF7.2* regulated tiller bud outgrowth under the different nitrate concentration. **a-e** Comparison of tiller buds among WT, OX lines, Ri lines and *osnpf7.2* under the 0.2 mM NaNO_3_, 2 mM NaNO_3_, 4 mM NaNO_3_, 6 mM NaNO_3_ and 8 mM NaNO_3_, respectively. White arrows indicated tiller buds, bar = 1 mm. **f-j** Statistical analysis of the tiller buds length among WT, OX lines, Ri lines and *osnpf7.2* under the 0.2 mM NaNO_3_, 2 mM NaNO_3_, 4 mM NaNO_3_, 6 mM NaNO_3_ and 8 mM NaNO_3_, respectively. DAG was the abbreviation of days after germination. Values in **f-j** are shown as mean ± SD (*n* = 20) from three replicates; “*”, “**” and “***” indicated significant differences at *P* < 0.05, *P* < 0.01 and *P* < 0.001, respectively

We further investigated the regulatory effort of *OsNPF7.2* on rice tillering by analysing of the development of tiller buds in WT, OX lines, Ri lines, and mutant *osnpf7.2* grown under different nitrate concentrations (0.5–8 mM). Tiller buds grew more rapidly in OX lines than in WT under all nitrate concentrations with this phenomenon observed continuously for 34 days after germination (DAG); tiller buds growth was slower in line Ri and mutant *osnpf7.2* than in WT (Fig. 2a-e). OX lines had significantly longer tiller buds than WT when plants were treated with 0.5 mM nitrate at 19–34 DAG (Fig. 2f). However, no significant difference in buds length between line OX and WT was observed under high nitrate concentrations (4–8 mM) at 19 DAG (Fig. 2h-j). OX lines had longer tiller buds than WT after 27 DAG at all nitrate concentrations, and the maximum length of tiller buds in OX lines were found at 34 DAG in plants treated with 8 mM nitrate (Fig. 2j). Significantly shorter tiller buds were observed in Ri lines than in WT (Fig. 2f-j). Based on these results, it could be concluded that elevated expression of *OsNFP7.2* promoted rice tiller bud outgrowth, especially under high nitrate concentrations, between 19 and 34 DAG.

### Elevated expression of *OsNPF7.2* benefits rice seedling growth and root development

**Fig. 3:**
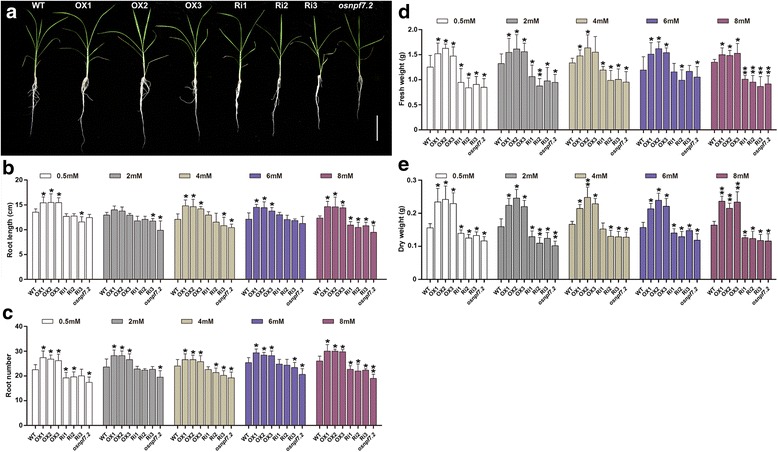
Altered expression of *OsNPF7.2* functioned on rice seedling growth at different nitrate supply. **a** Phenotypic analysis of seedlings at 31 DAG among WT, OX lines, Ri lines and *osnpf7.2* under the 8 mM NaNO_3_ supply, respectively. Bar = 10 cm. **b-e** Statistical analysis of root length (**b**), root number (**c**), fresh weight (**d**) and dry weight (**e**), respectively, among WT, OX lines, Ri lines and *osnpf7.2* under the 8 mM NaNO_3_ supply. Values in **b-e** are shown as mean ± SD (n = 20) from three replicates; “*” and “**” indicated significant differences at *P* < 0.05 and *P* < 0.01, respectively

Next, we investigated the effect of up-regulation of *OsNPF7.2* on rice seedling growth and development in hydroponic cultures under different nitrate concentrations. Seedlings of OX lines under 8 mM NaNO_3_ conditions produced stronger culms than seedlings of WT (Fig. 3a). However, Ri lines and mutant *osnpf7.2* showed the opposite result (Fig. 3a). Root morphology is important for plant to optimize N absorption from the soil through its responses to nitrates (Hachiya and Sakakibara 2017), we examined root development in *OsNPF7.2* transgenic lines. In Ri lines and mutant *osnpf7.2*, root growth was inhibited resulting in their roots were shorter than those of WT seedlings (Fig. 3a-b). The comparison of root number revealed that elevated expression of *OsNPF7.2* caused a significant increase at all nitrate concentrations, while down-regulated expression of *OsNPF7.2* result in a significant decrease of root number at 8 mM NaNO_3_ (Fig. 3c). Compared with those of WT, the fresh weight and dry weight of OX lines were significantly increased (Fig. 3d-e). These results demonstrated that genetically modification of *OsNPF7.2* could significantly influence rice root development.

We also cultured the different lines under 4 mM (NH_4_)_2_SO_4_, and found no significant difference in root length among WT, OX lines, Ri lines, and mutant *osnpf7.2* at the seedling stage (40 DAG, Additional file 1 Figure S1b and d). However, root length of OX lines exceeded that of WT when seedlings were treated with 8 mM NaNO_3_, whereas root length of the seedlings with down-regulated *OsNPF7.2* expression decreased (Additional file 1 Figure S1a and c). These results indicated that *OsNPF7.2* transgenic seedlings responded to environmental nitrate by altering root growth and development, but they did not respond to ammonium.

### Changes in expression of *OsNPF7.2* influence the rate of NO_3_^−^ influx and concentration

**Fig. 4:**
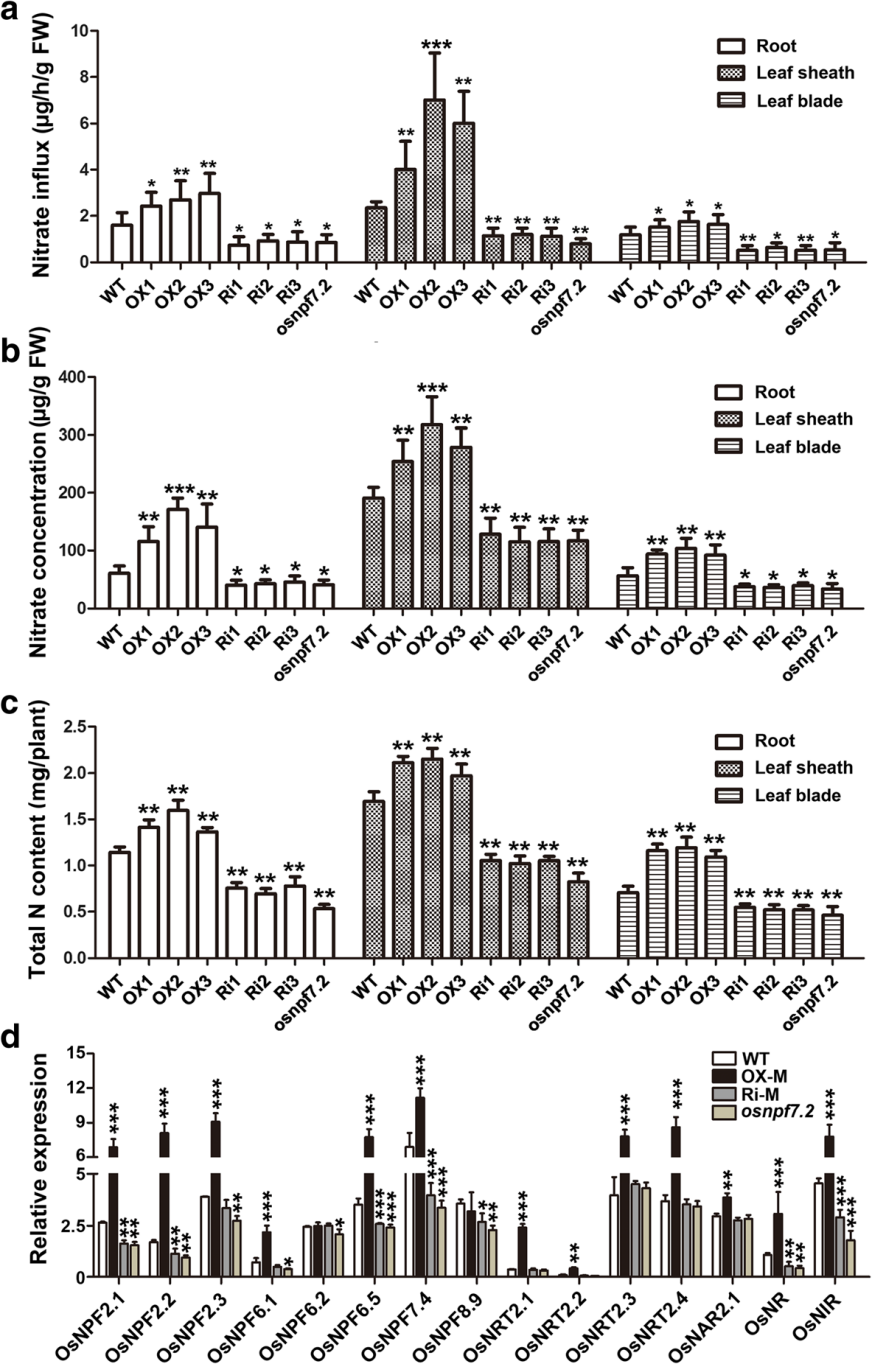
*OsNPF7.2* influenced NO_3_^−^ influx, NO_3_^−^ concentration and total nitrogen content among transgenic lines. **a** Analysis of NO_3_^−^ influx rate among WT, OX lines, Ri lines and *osnpf7.2* cultured under 6 mM NaNO_3_ supply. **b** NO_3_^−^ concentration of WT, OX lines, Ri lines and *osnpf7.2* cultured under 6 mM NaNO_3_ supply. **c** Comparison of total nitrogen content among WT, OX lines, Ri lines and *osnpf7.2* cultured under 6 mM NaNO_3_ supply. **d** Expression level of crucial genes involved in absorbing, transporting and assimilation of nitrate among WT, OX-M, Ri-M and *osnpf7.2* cultured under 6 mM NaNO_3_ supply. OX-M and Ri-M indicated that mixed equal-amount RNA which extracted from 10 seedlings’ tiller buds of each three OX lines and Ri lines, respectively. Date are shown as mean ± SD (n = 10) from three replicates; “*”, “**” and “***” indicated significant differences at P < 0.05, P < 0.01 and P < 0.001, respectively

Seedlings with various expression lines were treated under nitrogen starvation conditions for a week, and then were cultured with a solution containing 8 mM nitrate for 24 h. The amount of NO_3_^−^ obsorbed by seedlings was then measured. The rate of NO_3_^−^ influx into roots of OX lines was higher than that of WT (Fig. 4a), indicating that elevated expression of *OsNPF7.2* enhanced nitrate uptake by roots. In OX lines, we also detected a higher rate of NO_3_^−^ influx into the leaf sheath and leaf blade, implying that elevated expression of *OsNPF7.2* promoted the translocation of NO_3_^−^ from roots to leaf sheath (Fig. 4a). Besides, we measured nitrate concentration of root, leaf sheath and leaf blade in the seedlings with different expression lines. The detected nitrate concentration was consistent with the rate of NO_3_^−^ influx in different lines (Fig. 4b). Total nitrogen concentrations in root, leaf sheath, and leaf blade did not differ significantly among WT, OX lines, Ri lines, and mutant *osnpf7.2* (data not shown). However, total nitrogen content in those lines with up-regulated expression became higher than that in WT, and repression lines exhibited lower total nitrogen content compared to WT (Fig. 4c). These results demonstrated that over-expression of *OsNPF7.2* promoted translocation of nitrate from roots to leaf sheath, and enhanced nitrate influx and concentration.

We detected the effect of *OsNPF7.2* various expression lines on expression levels of 18 genes, including low-affinity nitrate transporters, high-affinity nitrate transporters, nitrate and ammonium assimilation genes. The expression level of 7 NPF genes including *OsNPF6.5* in OX lines was increased compared to that in WT (Fig. 4d). Two of the 18 genes, *OsNPF6.5* and *OsGS1;2*, were reported to promote tiller bud outgrowth and the increase in rice tiller number (Hu et al. 2015; Ohashi et al. 2017). Expression of both *OsNPF6.5* and *OsGS1;2* was up-regulated in OX lines and down-regulated in Ri lines and mutant *osnpf7.2* (Additional file 2 Figure S2). Based on these results, it could be concluded that elevated expression of *OsNPF7.2* promotes nitrate uptake and assimilation by regulating other nitrate-related transporters and enzymes.

### *OsNPF7.2* regulates cell proliferation in tiller bud by coordinating cytokinin and strigolactone pathways

**Fig. 5:**
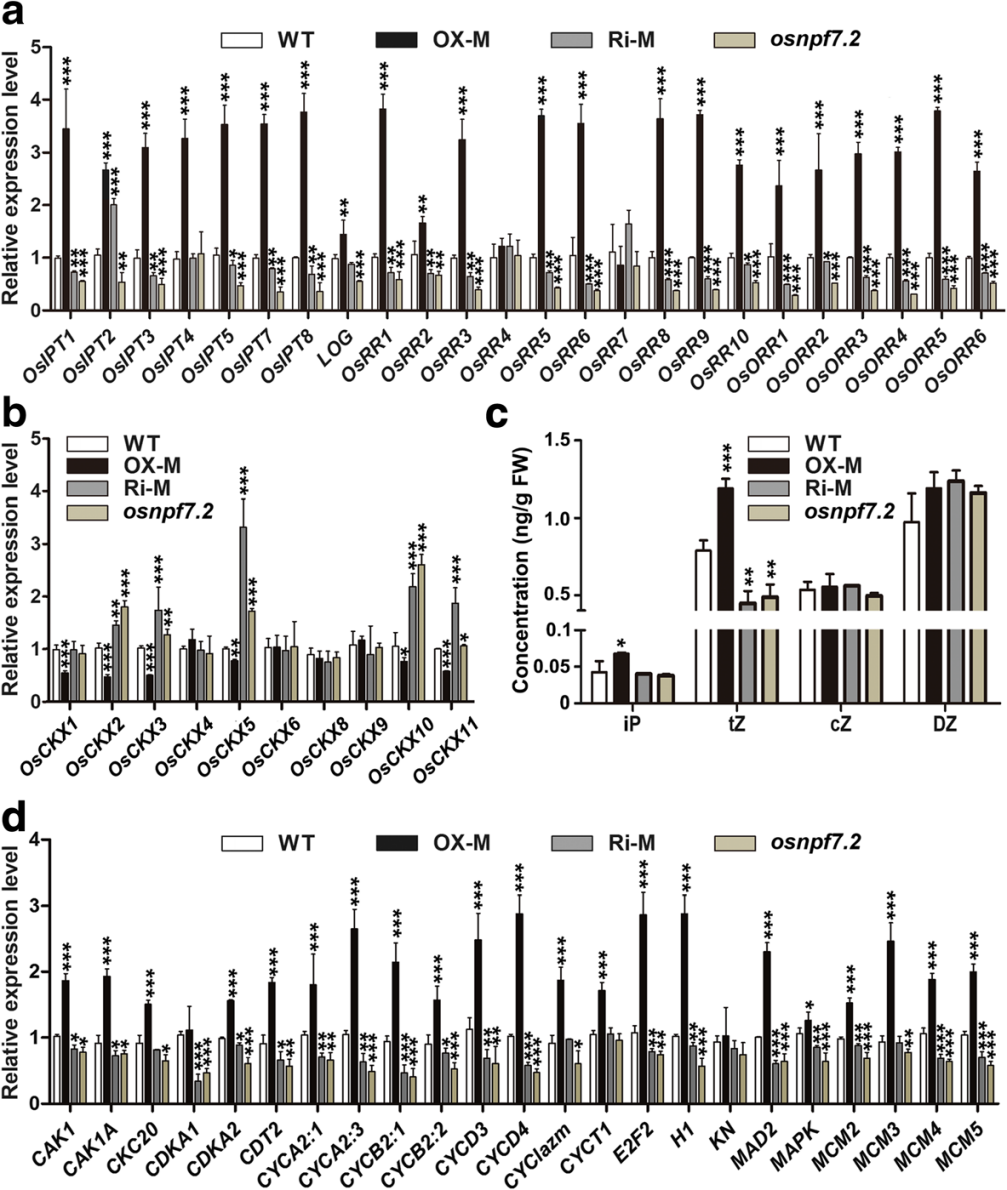
Altered expression of OsNPF7.2 regulated CKs concentration and cell cycle. **a** Expression level of rice genes involved in cytokinin synthesis and response in tiller buds of seedlings at 34 DAG among WT, OX-M, Ri-M and *osnpf7.2*. **b** Expression of 10 *CKXs* at 34 DAG among WT, OX-M, Ri-M and *osnpf7.2*. **c** CK free base concentration in seedling tiller basal portion at 34 DAG among WT, OX-M, Ri-M and *osnpf7.2*. **c** Comparison of genes involved in cell cycle in tiller buds of seedlings at 34 DAG among different transgenic lines. OX-M and Ri-M in (**a**)-(**b**) and (**d**) was identical to that in fig. 4d. OX-M and Ri-M in (**c**) indicated tiller basal portion (about 0.5 cm) mixed from 30 seedlings of each OX lines and Ri lines, respectively. Date are shown as mean ± SD from three replicates; “*”, “**” and “***” indicated significant differences at *P* < 0.05, *P* < 0.01 and *P* < 0.001, respectively

Shoot branching (tiller) is regulated by plant hormones, particularly cytokinins (CKs) and strigolactones (SLs). It was reported that CKs promote tillering in rice, whereas SLs inhibit it (Leyser 2003; Ferguson and Beveridge 2009; Hayward et al. 2009; Shimizu-Sato et al. 2009; Xu et al. 2015). To investigate the possible interaction between nitrogen and plant hormones, we measured the expression levels of the important genes responding to CK and SL pathways in the rice tiller bud with various transgenic lines. Over-expression of *OsNPF7.2* resulted in the up-regulated expression of *OsIPTs*, *LONELY GUY* (*LOG*), and CK-response regulators (*OsARRs*/*OsRRs*) (Fig. 5a). Levels of cytokinins were regulated through the irreversible oxidative cleavage of the N^6^-side chain by CYTOKININ DEHYDROGENASE/OXIDASE (CKXs) (Zurcher and Muller 2016). Based on their report, we measured gene expression of 10 *CKXs*, and found half of *OsCKXs* exhibited lower expression level in OX lines compared to that in WT, whereas higher expression level in both Ri lines and mutant than that in WT (Fig. 5b). Additionally, we also detected the content of four CKs (iP, tZ, cZ and DZ) in tiller basal portion among different genetically modified lines. Compared to WT, OX lines exhibited a significant increase in the concentration of iP and tZ, while Ri lines and mutant showed a little but not significant decrease in the concentration of iP and a significant decrease in the concentration of tZ (Fig. 5c). These results suggested that altered expression of *OsNPF7.2* controlled tiller bud outgrowth possibly by regulating CKs content in the tiller bud. Cytokinins function mainly by stimulating cell division and growth and by promoting cell differentiation as well (Zurcher and Muller 2016). However, cell proliferation is strictly controlled by the major regulators: cyclin-dependent kinases (CDKs) and their regulatory partner cyclins (Yamaguchi et al. 2003). We measured the expression levels of selected genes involved in cell cycle. Significant up-regulation of *OsNPF7.2* was associated with an increased expression level of *CDKs* and cyclin genes such as *CYCAs*, *CYCBs* and *CYCDs*. By contrast, Ri lines and mutant *osnpf7.2* showed decreased expression of these genes (Fig. 5d). We also found that the expression patterns of four minichromosome maintenance genes (*MCM2*, *MCM3*, *MCM4* and *MCM5*) were similar to those of cyclin genes in the transgenic lines (Fig. 5d). These results indicated that elevated expression of *OsNPF7.2* promoted tiller bud growth possibly by accelerating plant cell proliferation.

**Fig. 6:**
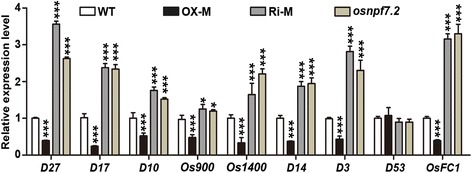
Expression of genes involved in strigolactone biosynthesis, perception and signalling pathway. Genes’ expression involved in SL biosynthesis (*D27*, *D17*, *D10*, *Os900* and *Os1400*), perception (*D14* and *D3*), and signalling (*D53*) were detected among different genetical modified lines. OX-M and Ri-M was same to that in fig. 4d. Date are shown as mean ± SD from three replicates; “*” and “***” indicated significant differences at *P* < 0.05 and *P* < 0.001, respectively

SL biosynthesis (*D27*, *D17*, *D10*, *Os900* and *Os1400*), perception (*D14* and *D3*), and signalling (*D53*) were reported to have participated in the regulation of tiller bud outgrowth (Jiang et al. 2013; Zhou et al. 2013; Liang et al. 2014; Zhang et al. 2014). These SL synthesis and signalling genes were also detected in tiller bud in the different transgenic lines. The genes (*D27*, *D17*, *D10*, *D14*, and *D3*) were down-regulated in OX lines but up-regulated in Ri lines (Fig. 6). Besides, two members of CYP711 enzymes (*Os900* and *Os1400*, Zhang et al. 2014) showed similar expression pattern to that of *D27*, *D17*, *D10*, *D14*, and *D3* (Fig. 6). However, expression level of *D53*, a repressor of SL signalling, exhibited no significant differences among WT, OX lines, Ri lines, and mutant (Fig. 6). It was reported that the interaction between OsMADS57 and OsFC1/OsTB1 targets *D14* to control the outgrowth of tiller bud in rice (Guo et al. 2013). We compared the expression level of *OsFC1* in tiller bud in the various transgenic lines and found that *OsFC1* was down-regulated in OX lines and was up-regulated in Ri lines and mutant (Fig. 6). SLs, as most of the germination stimulants identified so far, function on stimulating germination of root parasitic plants such as witchweeds (*Striga* spp.) and broomrapes (*Orobanche* and *Phelipanche* spp., Yoneyama et al. 2010). We performed germination assay of *Orobanche Cumana* to estimate SLs level, and found that the germination rate of *Orobanche Cumana* seeds was higher when the seeds were treated with root exudates extracting from Ri lines and mutant *osnpf7.2* than that from WT, and the opposite results were found when the seeds were treated with root exudates from OX lines (Additional file 3 Figure S3). These results indicated that altered expression of *OsNPF7.2* might influence SL biosynthesis, which in turn influenced perception and signalling in rice tiller bud, therefore controlled rice tillering.

**Fig. 7:**
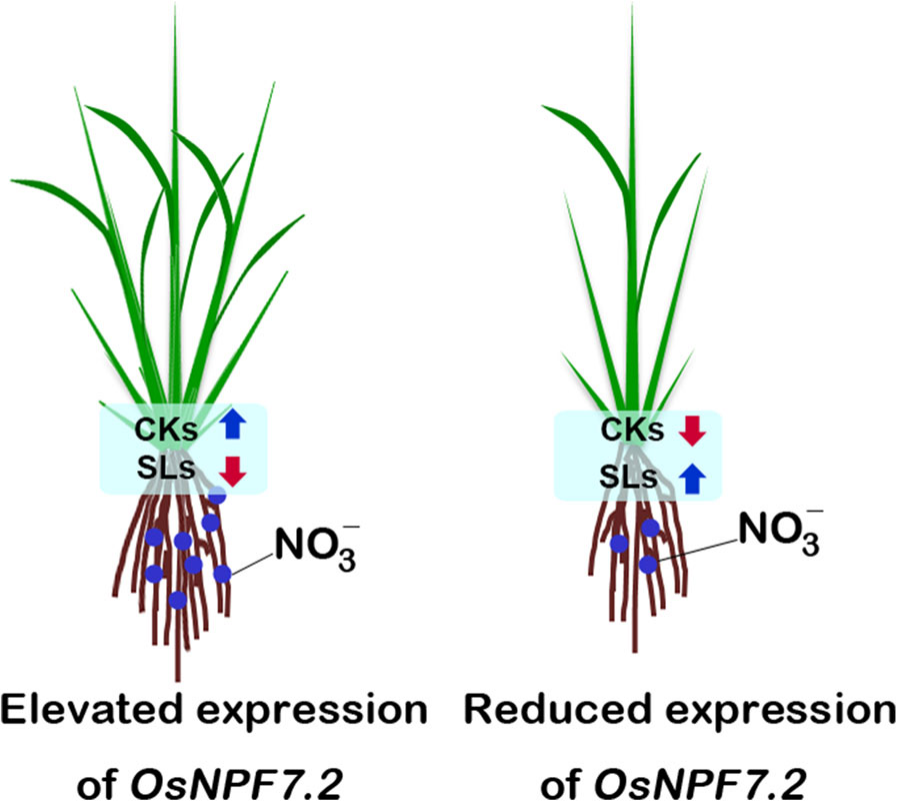
The proposed model of *OsNPF7.2* regulating rice tillering. Blue circles suggested nitrate, and the different number of circles indicated the different nitrate concentration between overexpression lines and repression lines. CKs and SLs were abbreviations of cytokinins and strigolactones

Based on these results, we propose a model in which altered expression of *OsNPF7.2* participates in CK and SL pathways to modify rice tillering (Fig. 7). In OX lines, elevated expression of *OsNPF7.2* is capable of enhancing CK accumulation and inhibiting SL accumulation. Hence, cell division in the tiller bud is promoted, which is favourable for rice tillering. However, down-regulation of *OsNPF7.2* induces the opposite effects. Additionally, we suggest that *OsNPF7.2* coordinates CK and SL pathways, and further regulates tiller bud, eventually controls rice tillering.

## Discussion

### *OsNPF7.2* positively regulates rice tiller number and grain yield

Nitrogen is a crucial determinant of plant growth and grain yield (Hachiya and Sakakibara 2017; Li et al. 2017). Plants make use of transporters to take up nitrogen from the soil via the roots and transport it to other organs. Thus, the coordinated expression of transporter genes is essential to meet the plant growth’s requirements for nitrogen. Up till now, only a few nitrate transporters of NPF family (*OsNPF8.9*, *OsNPF2.2*, *OsNPF2.4*, *OsNPF6.5*, and *OsNPF7.2*) have been characterized in rice (Lin et al. 2000; Li et al. 2015; Xia et al. 2015; Hu et al. 2016). Of these, only *OsNPF6.5* regulates rice tiller number and promotes grain yield (Hu et al. 2015). Our study revealed that elevated expression of *OsNPF7.2* significantly enhances tiller number, whereas repressed expression causes a reduction in tiller number (Fig. 1). However, previous study only reported the retarded growth of rice root of plant mutant *osnpf7.2* (Hu et al. 2016). Rice tiller number was reported to be one of the most important agronomic traits determining panicle number and grain yield (Li et al. 2003; Xing and Zhang 2010). The increased number of panicles resulted in a larger number of filled grain per plant in OX lines than in WT, which gave rise to an improved rice grain yield per plant (Fig. 1r-t and v). Interesting, 1000-grain weight among all genetically modified lines exhibited slight decreased compared with that in WT (Fig. 1u), which indicated that appropriate expression of *OsNPF7.2* might be beneficial to increase grain weight. This study suggested that *OsNPF7.2* may be useful to culture high-yield rice varieties.

### *OsNPF7.2* influences NO_3_^−^ influx and concentration, and tiller bud growth

One previous study reported that vacuolar-membrane-localized OsNPF7.2 could transport nitrate at a low affinity (Hu et al. 2016). Our study showed that over-expression of *OsNPF7.2* enhanced the rate of NO_3_^−^ influx into roots, increased NO_3_^−^ concentration in root, and promoted the translocation of nitrate from roots to leaf sheath (Fig. 4a-c), indicating that elevated expression of *OsNPF7.2* contributes to nitrate allocation between roots and shoots. Recently, three NRT1/NPF family members (NPF5.11, NPF5.12 and NPF5.16) in *Arabidopsis* were reported to be localized at vacuolar membrane and to play a possible role in modulating nitrate allocation between roots and shoots (He et al. 2017).

*NRT1.1B–indica* allele was reported to increase tiller number per plant, and to enhance grain yield per plant (Hu et al. 2015). Our study found that up-regulated expression of *OsNPF7.2* significantly enhanced tiller bud growth, and that down-regulated expression of *OsNPF7.2* impaired tiller bud development (Fig. 2), suggesting that the enhanced translocation of nitrate into leaf sheath in OX lines might contribute to faster growth of tiller bud determining rice tiller number. In addition, the biomass of OX lines was higher than that of WT (Fig. 3d-e), which might mainly be attributed to the increased tiller number at vegetative stage. Therefore, it can be concluded that elevated expression of nitrate transporter *OsNPF7.2* not only promoted rice tillering at vegetative stage, but also played a potentially important role in increasing rice grain yield at reproductive stage.

### Elevated of *OsNPF7.2* promotes cell division and rice tiller formation through cytokinin and strigolactone pathways

It has been reported that elevated CK level might promote tiller bud outgrowth (Turnbull et al. 1997). Our study indicated that the expression of such CK crucial genes as *IPTs* and *LOG* was higher in OX lines than in WT, suggesting that CKs probably produced in larger amounts in OX lines than in WT; however, the opposite result was found in Ri lines and in mutant (Fig. 5a). Furthermore, the expression pattern of CK response genes (*OsARRs* and *OsRRs*) was similar to *IPTs* expression pattern in transgenic lines. Moreover, the content of iP and tZ was higher in OX lines than in WT (Fig. 5c), which indicated that over-expression of *OsNPF7.2* enhanced CK accumulation in tiller bud.

Nitrogen uptake, assimilation, and recycling in plant roots were reported to determine plant development and productivity (Yamaya and Kusano 2014). However, numerous plant developmental processes such as root meristem specification, vascular development, and shoot and root growth, are determined by CKs (Zurcher and Muller 2016). In addition, CKs are key phytohormones for cell division and growth (Riou-Khamlichi et al. 1999). This study illustrates that elevated expression of *OsNPF7.2* promotes up-regulation of crucial genes in cell cycle (Fig. 5d), which indicated that over-expression of *OsNPF7.2* might promote cell division. To explore the regulatory mechanism of *OsNPF7.2*, we analyse expression of the major regulators in cell cycle, namely, cyclin-dependent kinases (CDKs) and their regulatory partner cyclins. The analysis shows that up-regulation of *OsNPF7.2* significantly increases the expression level of *CDKs*, while down-regulation of *OsNPF7.2* reduces the level of expression of *CDKs* (Fig. 5c). Based on these results, we conclude that altered expression of *OsNPF7.2* controls rice tillering by regulating CK contents, and further regulating the cell cycle in tiller bud.

Recently, SLs have been reported to be important phytohormones inhibiting tiller bud outgrowth in various plant species (Gomez-Roldan et al. 2008; Umehara et al. 2008). Reduction of SL production, perception, and signalling results in faster outgrowth of tiller bud (Domagalska and Leyser 2011; Ruyter-Spira et al. 2013). This study found that *D27*, *D17*, *D10*, *Os900*, *Os1400*, *D14*, *D3*, and *OsFC1* were down-regulated in OX lines but up-regulated in Ri lines and mutant, compared to WT (Fig. 6). OX lines displayed reduced SL biosynthesis, perception, and signalling, whereas Ri lines and mutant showed increased SL signalling. Based on these results, we construct a model of altered expression of *OsNPF7.2* function in rice tillering, in which over-expression of *OsNPF7.2* enhances CK levels but might inhibit SL pathway, which results in increased tillering (Fig. 7). However, the reduced tiller number in Ri lines and mutant *osnpf7.2* might have resulted from weaker CK and stronger SL signalling in their tiller bud.

## Conclusions

In this study, we constructed transgenic lines with different expression level of *OsNPF7.2* and found that elevated expression of *OsNPF7.2* contributed to the enhancement of NO_3_^−^ influx rate and the increase of nitrate concentration in over-expression lines. Importantly, *OsNPF7.2* positively regulated tiller bud outgrowth, probably by coordinating CK and SL pathways in plant cells.

## Methods

### Generation of transgenic rice lines

To construct *OsNPF7.2-*overexpressing lines, a 1726-bp *OsNPF7.2* cDNA was inserted downstream of the *35S* promoter in pCAM1301 using BglII and AflII, to produce the *p35S*-*OsNPF7.2* plasmid. To generate the *OsNPF7.2*-RNAi lines, two 323-bp fragments of *OsNPF7.2* cDNA were amplified and inserted downstream of the *Ubi-1* promoter in vector pTCK303 (Wang et al. 2004). All of the constructed plasmids were transferred into *japonica* rice variety ‘Zhonghua 11’ (ZH11) by the *Agrobacterium*-mediated transformation method (Hiei et al. 1997). Homozygous T2 generation of each transgenic lines screened with hygromycin at final concentration of 50 mM for a week were chosen for further studies. Mutant *osnpf7.2* in ZH11 background was obtained from the Rice Mutant Database of Huazhong Agricultural University (http://rmd.ncpgr.cn/), which was the same to that used in the previous study (Hu et al. 2016). The corresponding primers are listed in Additional file 4 Table S1.

### Plant cultivation and agronomic traits analysis

For basic agronomic traits analysis, rice plants were grown in the paddy field from June to October at the rice experimental station of the Wuhan Institute of Bioengineering. Ten plants at a spacing of 16.5 cm × 26.5 cm were planted in a row and 5 rows of each line were planted. At reproductive stage, 10 plants of each lines were randomly chosen to detect agronomic traits. The grain number per panicle was measured as the total number of grains per plant divided by the number of panicles per plant. The 1000-grain weight was calculated as the weight of the total grains per plant and divided by the grain number, then converted to 1000-grain weight. Grain yield was measured as the weight of total grains per plant.

To analyse *OsNPF7.2* expression function in seedling growth and development under different nitrogen conditions, ZH11, OX, Ri, and *osnpf7.2* seedlings at 7 DAG were cultured in basic nutrient solution (pH = 5.8) for a week. The composition of the basic solution was as follows: 1 mM NH_4_NO_3_, 0.32 mM NaH_2_PO_4_, 0.51 mM K_2_SO_4_, 1 mM CaCl_2_, 1.65 mM MgSO_4_, 8.9 μM MnSO_4_, 0.5 μM Na_2_MoO_4_, 18.4 μM H_3_BO_3_, 0.14 μM ZnSO_4_, 0.16 μM CuSO_4_ and 40 μM FeSO_4_. Then seedlings at 14 DAG were transferred to basic nutrient solutions supplemented with the following sole nitrogen source: 0.5 mM NaNO_3_, 2 mM NaNO_3_, 4 mM NaNO_3_, 6 mM NaNO_3_, 8 mM NaNO_3_ and 4 mM (NH_4_)_2_SO_4_. Each nutrient solution was renewed every three days. Daytime conditions in the greenhouse were 32 °C, with light from a sodium lamp (400 W) for 14 h; night-time conditions were 25 °C, and dark for 10 h. At 34 DAG, root length, root number, fresh weight and dry weight of each lines were measured. Besides, tiller buds of different lines (34 DAG) were obtained to detect the expression level of *OsNPF7.2* and other phytohormone-related genes.

### Measurement of nitrate influx, nitrate concentration, and total nitrogen content

To analyse the nitrate influx, nitrate concentration and total nitrogen content, ten seedlings at 7 DAG of ZH11, OX, Ri, and *osnpf7.2* were cultured in basic nutrient solution for a week. They were then placed in basic nutrient solution without nitrogen for a week for nitrogen-starvation treatment. The nitrogen-starved seedlings were transferred to culture solution containing 8 mM nitrate for 24 h. Free NO_3_^−^ content analysis was carried out by homogenizing plant tissues in cold extraction buffer [50 mM Tris-HCl (pH 7.0), 10 mM imidazole, and 0.5% (*w*/*v*) β-mercaptoethanol]. The suspension was centrifuged at 12,000 rpm for 30 min and the supernatant was collected. Free NO_3_^−^ content was determined from a standard curve of KNO_3_ (Cai et al. 2009). NO_3_^−^ influx was calculated as the difference in NO_3_^−^ content between the 8 mM nitrate-treatment and nitrate-starved plants in an hour. Total nitrogen content was determined using the semi-micro Kjeldahl method using a nitrogen analyser (Smart Chem 200, Westco, Italy). Three replicates of each assay were performed.

### RNA isolation and qRT-PCR

Total RNA was extracted from tiller buds using TRIzol reagent (Invitrogen, Beijing, China). First-strand cDNA was synthesized using random primers and MLV reverse transcriptase (TaKaRa Bio, Beijing, China). qRT-PCR reaction solution was prepared in a total volume of 20 μL, containing 2 μL of the cDNA, 0.2 mM of each primer, and 10 μL of 2 × SYBR green PCR master mix (Takara Co. Ltd., http://www.takarabiomed.com.cn/). Quantitative real-time PCR was performed using SYBR Green mix (TaKaRa Bio, Beijing, China) and the 7500 RT qPCR system (Applied Biosystems, Foster City, CA, United States). The rice *Actin* gene (LOC_Os03g50885) was used as the internal control, and three technical replicates were performed for each sample. Expression level was calculated using the relative quantification method (Carleton 2011). The primers used for qPCR are listed in Additional file 4.

### Extraction of root exudates and germination assay of *Orobanche cumana* seeds

Rice seedlings at 7 DAG were cultured in basic nutrient solution with 6 mM NaNO_3_ supply for a month, then root exudates of ZH11, OX lines, Ri lines and *osnpf7.2* seedlings were extracted using a modified method (Chen et al. 2017). The shoots (5 cm above the roots) were excised with a razor, and the xylem sap was collected for 12 h after decapitation of the shoots. Root exudates were then diluted with distilled water for 10 times to stimulate *Orobanche cumana* seeds germination.

Germination assay was performed according to Ma et al. (2005). *Orobanche cumana* seeds (20–40 seeds) were incubated on 8 mm moist glass-fiber filter paper at 30 °C for 7 days, and then 30 μl diluted root exudates were applied to glass-fiber filter paper to stimulate seeds germination. Germination of the treated seeds was recorded after incubated at 30 °C for another week. Three replicates of each assay were performed and germination data were statistically analyzed using SPSS software.

### Determination of CKs concentration

The tiller basal portion (about 0.5 cm) from 30 seedlings of each OX line (OX1, OX2 and OX3) at 34 DAG were mixed, which were named as OX-M. Ri-M indicated the mixed tiller basal portion from 30 seedlings of each RNA interference lines (Ri1, Ri2 and Ri3) at 34 DAG. Then CKs content were measured by MetWare (http://www.metware.cn/) based at ABSciexQTRAP®4500LC-MS/MS platform among different genetically modified lines. Three replicates of each assay were performed.

## Additional files

Additional file 1 **Figure S1.** *OsNPF7.2* responded to nitrate specially, not to (TIFF 4917 kb) ammonium. **a** Phenotypic analysis of seedlings (40 DAG) of transgenic lines.cultured under the 8 mM NaNO_3_. Bar = 10 cm. **b** Seedlings (40 DAG) of WT, OX.lines, Ri lines and *osnpf7.2* cultured under the 4 mM (NH_4_)_2_SO_4_. Bar = 10 cm. **c-d.**Statistical analysis of root length of transgenic lines cultured under the 8 mM. NaNO_3_ and 4 mM (NH_4_)_2_SO_4_, respectively. Date are shown as mean ± SD (*n* = 10). From three replicates; “*” and “**” indicated significant differences at *P* < 0.05 and *P*.< 0.01, respectively.

Additional file 2 **Figure S2.** Transcript abundance of two glutamine synthetase *GS1;2* and *GS2* in tiller buds among transgenic lines. Date are shown as mean ± SD from three replicates; “*”, “**” and “***” indicated significant differences at P < 0.05, P < 0.01 and P < 0.001, respectively. (TIFF 127 kb)

Additional file 3 **Figure S3.** Gemination rate of *Orobanche cumana* seeds. To estimated SLs levels among ZH11, OX lines, Ri lines and mutant *osnpf7.2*, root exudates of each line were applied to pre-incubated *Orobanche cumana* seeds. Date are shown as mean ± SD from three replicates; “*” indicated significant differences at *P* < 0.05. (TIFF 4595 kb)

Additional file 4 **Table S1.** Primers used in this study.

## Abbreviations

CK: Cytokinin
DAG: Days after germination
OX: Over-expression
OX-M: Over-expression lines mixed
Ri: RNA-interference
Ri-M: RNA-interference lines mixed
SL: Strigolactone
WT: Wild type
