# Supplementary figures and images for: Rice nitrate transporter OsNPF7.2 positively regulates tiller number and grain yield
Source: Rice (N Y). 2018 Feb 27;11:12. doi: 10.1186/s12284-018-0205-6 (PMC5826914; doi:10.1186/s12284-018-0205-6)

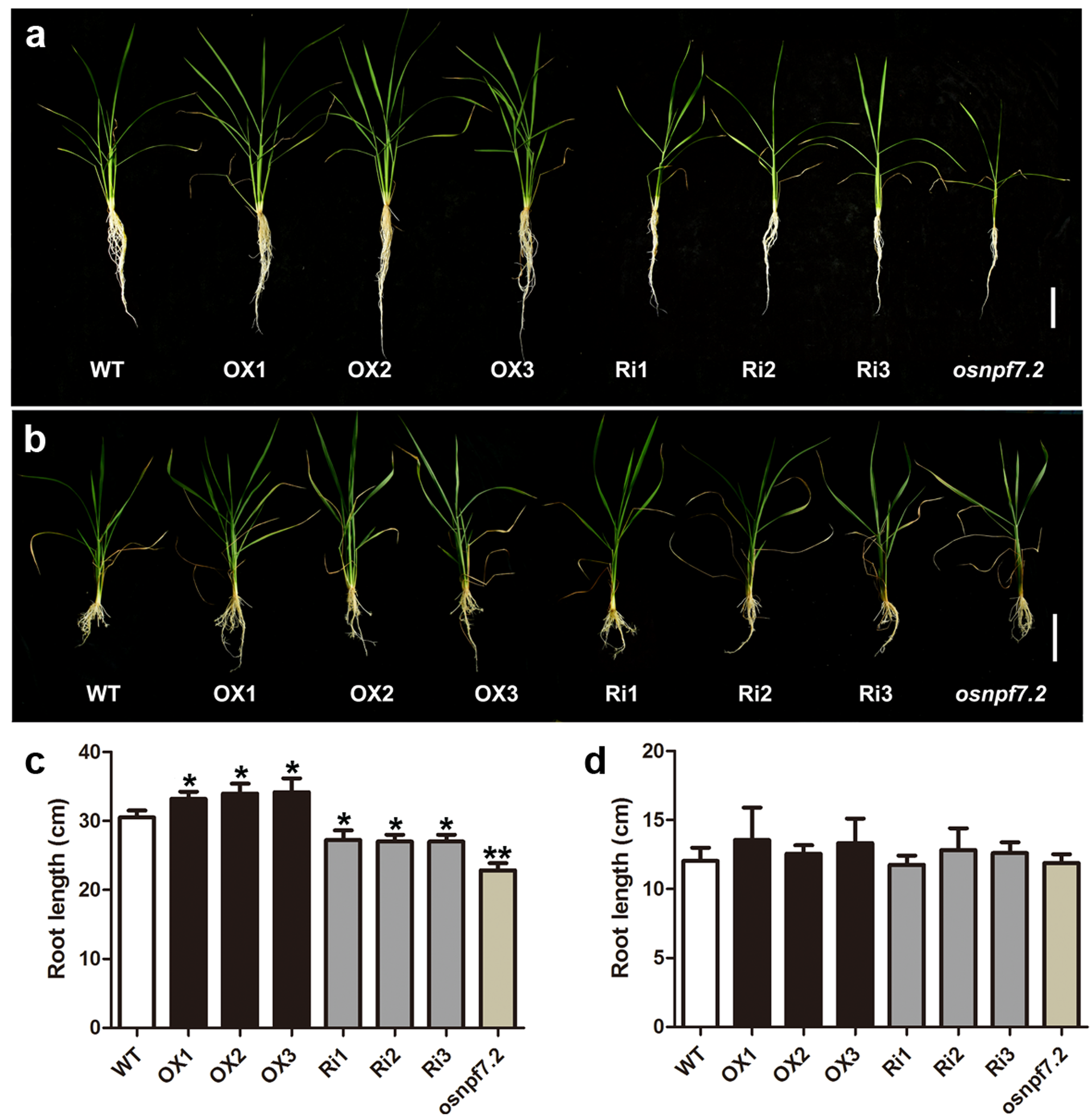

Supplement: Supplementary file 1 — Figure S1. OsNPF7.2 responded to nitrate specially, not to (TIFF 4917 kb) ammonium. a Phenotypic analysis of seedlings (40 DAG) of transgenic lines.cultured under the 8 mM NaNO3. Bar = 10 cm. b Seedlings (40 DAG) of WT, OX.lines, Ri lines and osnpf7.2 cultured under the 4 mM (NH4)2SO4. Bar = 10 cm. c-d.Statistical analysis of root length of transgenic lines cultured under the 8 mM. NaNO3 and 4 mM (NH4)2SO4, respectively. Date are shown as mean ± SD (n = 10). From three replicates; “*” and “**” indicated significant differences at P < 0.05 and P.< 0.01, respectively. [file 12284_2018_205_MOESM1_ESM.tif]

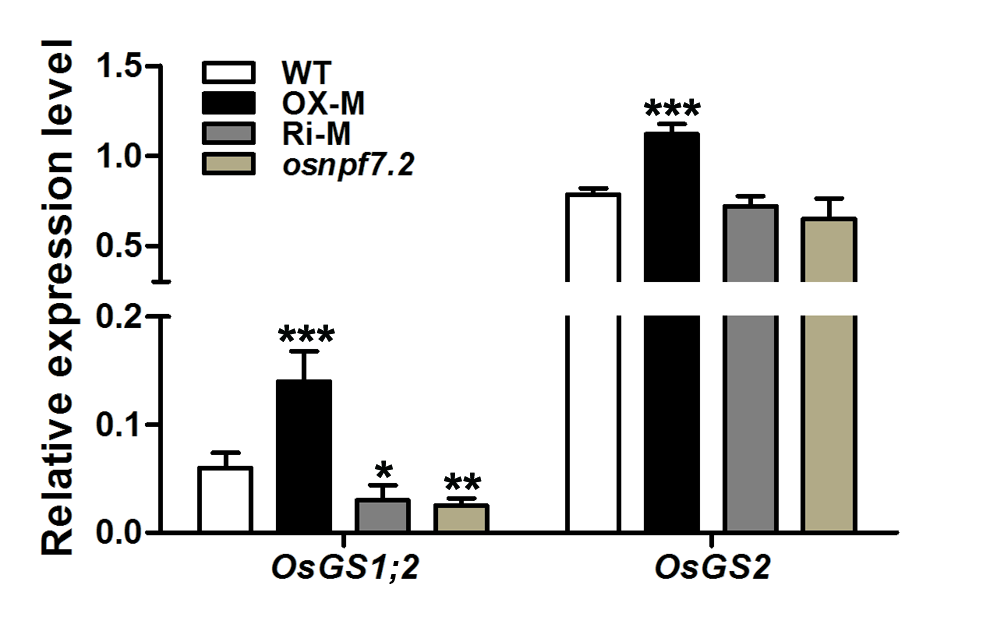

Supplement: Supplementary file 2 — Figure S2. Transcript abundance of two glutamine synthetase GS1;2 and GS2 in tiller buds among transgenic lines. Date are shown as mean ± SD from three replicates; “*”, “**” and “***” indicated significant differences at P < 0.05, P < 0.01 and P < 0.001, respectively. (TIFF 127 kb) [file 12284_2018_205_MOESM2_ESM.tif]

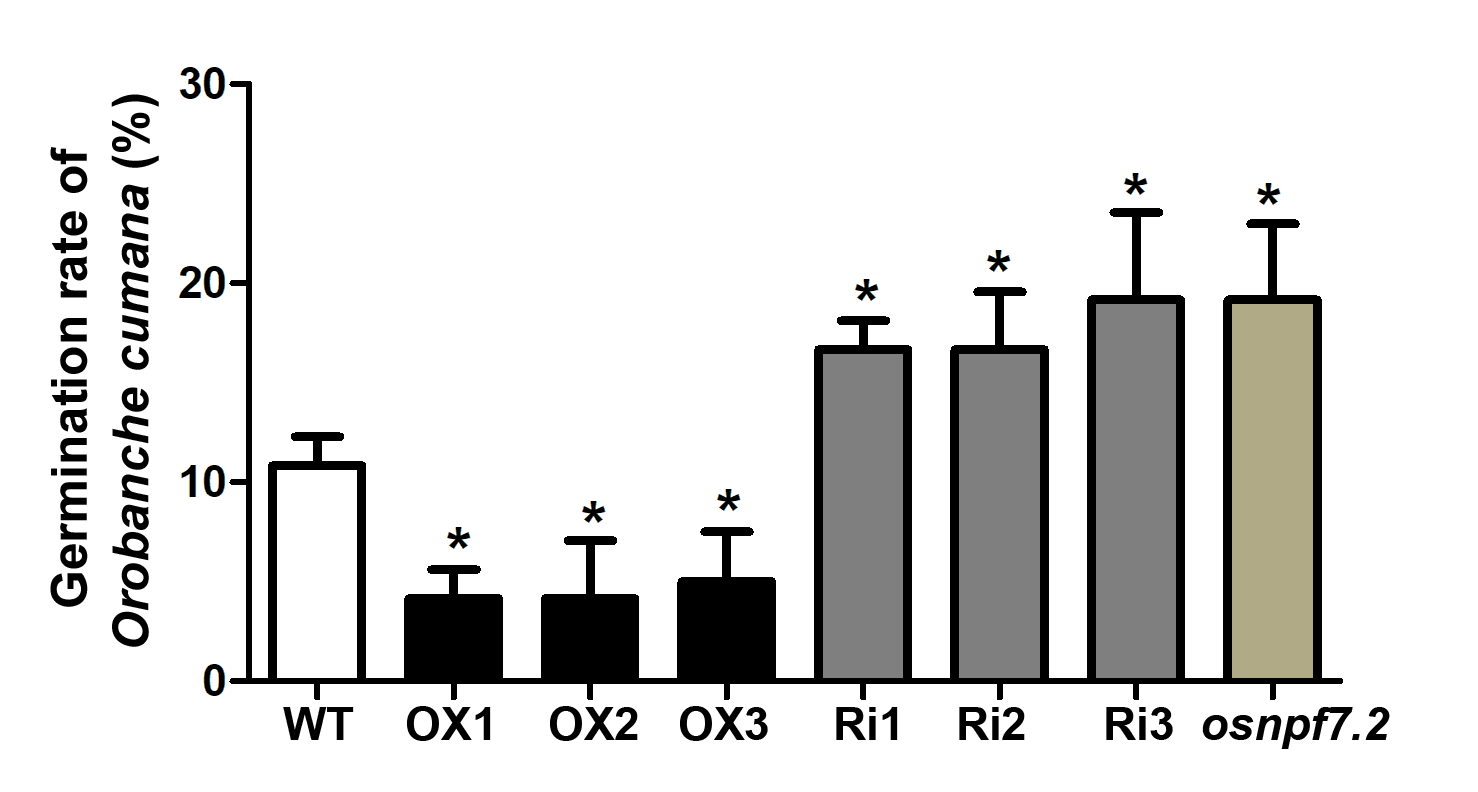

Supplement: Supplementary file 3 — Figure S3. Gemination rate of Orobanche cumana seeds. To estimated SLs levels among ZH11, OX lines, Ri lines and mutant osnpf7.2, root exudates of each line were applied to pre-incubated Orobanche cumana seeds. Date are shown as mean ± SD from three replicates; “*” indicated significant differences at P < 0.05. (TIFF 4595 kb) [file 12284_2018_205_MOESM3_ESM.tif]
