# Supplementary material for: Rice nitrate transporter OsNPF7.2 positively regulates tiller number and grain yield
Source: Rice (N Y). 2018 Feb 27;11:12. doi: 10.1186/s12284-018-0205-6 (PMC5826914; doi:10.1186/s12284-018-0205-6)
Supplement: Supplementary file 4 — Table S1. Primers used in this study. [file 12284_2018_205_MOESM4_ESM.docx]

| **Names** | **Forward sequence (5' - 3')** | **Reverse sequence (5' - 3')** |
| --- | --- | --- |
| OX-*NPF7.2* | AGATCTGTCATGGACGCCGGAGACGCCAT | CTTAAGGACGTCTCACGAGAGCACGGTCT |
| Ri-*NPF7.2*R | ACTAGT AGCGCGTCCAACGTCACGAC | GAGCTC GCGCTGTCGTCGTCGAACTG |
| Ri-*NPF7.2*F | GGTACC AGCGCGTCCAACGTCACGAC | GGATCC GCGCTGTCGTCGTCGAACTG |
| q*OsActin* | CGGTGTCATGGTCGGAAT | GCTCGTTGTAGAAGGTGT |
| q*OsNPF7.2* | TGCAAGTGCCACTCCTCAAGG | AGGACGGTCTCCAGGTACACCACC |
| q*OsNPF2.1* | AAGGGCGGGTGGATCACGTTCCCT | ACGCTGATGCAGCCGGAGACGAT |
| q*OsNPF2.2* | ACGGCGAACCTGCTGGTGTACC | TGAGGGTGAGCACAAGCATGCCGA |
| q*OsNPF2.3* | ATGGACGCAGCAGCAGCCAT | TGGTGCCGATGGTGCCCAGCTT |
| q*OsNPF6.1* | GGTGTTGGTGTTGTGGACATCC | AGGCCGTAGTAGGCCATCCT |
| q*OsNPF6.2* | GTCACCGACTTCATGGGCAC | TAGAGCACGGCCATCTGCAC |
| q*OsNPF6.5* | TGGCGATGGTGTTGCCGGAGA | AACAGCTCCGCCCCGAGGATCA |
| q*OsNPF7.4* | ACTCCAGGACGACGTCAGCCT | ACCAGGTTCGTGGCAATGCCGT |
| q*OsNPF8.9* | CATGGTTGCTTTAGGGTATGGT | GTGCAGAAATGGTAAAACCCCAG |
| q*OsNRT2.1* | CTACTACGGCTCCGAGTGGA | TGCTGGTCTCGGTCGCAGAA |
| q*OsNRT2.2* | CTACTACGGCTCCGAGTGGA | AAGGCCTTTCTCCATGACGA |
| q*OsNRT2.3* | GCGAGAAGGGTTTCAACGCGGCCA | ACCGCGACCTTATTGTCCGTGGCA |
| q*OsNRT2.4* | ACCAGCACTGGATGTCGCGGATCT | ACGAGCATGGCGCACGGGAGTA |
| q*OsNAR2.1* | CTTCGACGTCGCCGGGATCA | CTTGCGTTTGCAGCAAGACGAT |
| q*OsNR* | CCTGGAGAAGATGGGCTAT | GCACAACCATCCATCAATC |
| q*OsNIR* | CGAGGAGTAGGAACACAG | TGTCGTCTACTTTACAAGGA |
| *OsGS1:2* | TGTTTCTCCTCATCCCTGC | TCACAGTCCTCGCTTTGC |
| q*OsGS2* | GGAGCAGGCTGGTGTAGTGC | TCTCCCTGAATTGGTTTGGG |
| q*CAK1* | GACGGTCAGATTAGACGCAAGA | TCCAAAGGATGTCCACA |
| q*CAK1A* | GACCGACAAGGGTTTCAGCAT | CCAGCATGTTCAGGAAGATACAAT |
| q*CDC20* | TCGAATCACCTGTTTGTTGGC | TGGAGACAATCCAACGCAAAG |
| q*CDKA1* | GGTTTGGACCTTCTCTCTAAAATGC | AGAGCCTGTCTAGCTGTGATCCTT |
| q*CDKA2* | CGAGATTTGAAGCCCCAGAA | TCCGCGAGCTTCAATGAGTT |
| q*CDT2* | AACCGCACCAAACACTGGAA | GCAATTCACCATCTGCACTGG |
| q*CYCA2.1* | AGGTTGTCAAGATGGAGAGCGA | CGCTTTTTGTCTTCCTGGCA |
| q*CYCA2.3* | GTTTCGGTTGACGAGACGATGT | CGCTGCAAGGAACCTAGAACTG |
| q*CYCB2.1* | AAGTTTGGCCAGGAGTGAGCA | TCAAGAGCATCAGCGTCGAGA |
| q*CYCB2.2* | CTCAAGGCTGCACAATCTGACA | GCATTGACGGCTGGAATTTG |
| q*CYCD3* | CCTTCCACACTGACGGTACAGTT | TGCCGCTGCCAAATAGACA |
| q*CYCD4* | GCCATGGAGTTGATACATCCAA | CCAGTAGGGCTCCGTGGAAT |
| q*CYCIaZm* | CACTCTCAAGCACCACACTGGA | ACAACCCTCAGCTTGCTCTCAG |
| q*CYCT1* | GCATTTGTTGCAGCTCAAG | TCACCACTTCGCTGACTTATTG |
| q*E2F2* | TGTTGGTGGCTGCCGATAT | CGCCAGGTGCACCCTTT |
| q*H1* | GCAAGGCACCTGCAGCTT | AGGCAGCCTTTGTACAGATCCT |
| q*KN* | CACCAGCTTCAAGAGATCGTGA | CCGGAATTGAGACACAACTGC |
| q*MAD2* | GAGCCATGCATATTCGACGTG | GGTGTCGAAGGAATGCAGCTT |
| q*MAPK* | ACAGAGCAGCCGAATTTTGAGA | TTCAGCGAAGCTCACACTTGG |
| q*MCM2* | AAGTTGGCAAAAGATCCACGG | CCCCCAAACATAGCTAGTGCAA |
| q*MCM3* | TTCATGCGTCACTAAATGCGAG | TGAATCTGGAAGCCCAATGTTC |
| q*MCM4* | CCCGAATGCGATTCTCTGAA | ACCAGTGGCATGATCAGTTGC |
| q*MCM5* | AAGGAGAACTGCCTGTCCATGA | AGTGGCCTTAGCTTTCACCCTC |
| q*IPT1* | ACCAAGCCCAAGGTTATCTTCGTGC | TCGTCGGTGACCTTGTTGGTGATGA |
| q*IPT2* | AAGTCCAAGCTCGCCATCTCCATCG | GGTGACCTTGTTCGTGATGATGGGGA |
| q*IPT3* | AGGCGAACACGTGGAGTCTG | CCACCTTCAACTCCAGCACTCT |
| q*IPT4* | GTACGAGTGCTGCTTCCTCTGGGTC | CCAGATGCCCCTGGAGTAGTCGGGTG |
| q*IPT5* | CAGCGTCAGCAGGAGCATGGTAGCG | CGCGGCCGTGAACTCCTCGTCCGGG |
| q*IPT7* | AGGATACGAGGATGGTGGTGAT | CCGTCATAGAGCTGAATCTTGTC |
| q*IPT8* | CGAGGAGCTCGAGGAATACTTCGCG | TCTCGTCTATCGCCGCGTCGAGGCT |
| q*LOG* | ATCATCTCGGCTCCAACTGC | TCAGGATGAGGTGATCCTGG |
| q*OsRR1* | AGGATCAGCAGATGCATGAATG | GAGACGCTGTACGTCCTTGCTT |
| q*OsRR2* | ACGATCTTCTCAAAGCCATCAAG | TGAGAGGCTTAAGGATGAAATCCT |
| q*OsRR3* | CAGGGTTCGATCTCCTCAAGAG | CGAATTCTCCGACGACATTAGC |
| q*OsRR4* | TCTTCTGAGAATGTGCCTGCAA | GCTTGACAGGTTTCAGGAAGAACT |
| q*OsRR5* | ACCGAATGTGAGCATGATTATCA | CCTTGACCTTCTTCAGGAGTTCATA |
| q*OsRR6* | GTCCCCAACGTCAACATGATC | CACGTTCTCCGACGACATGAT |
| q*OsRR7* | TGCTCAAGAAGATCAAGGAATCG | GGCACGTTCTCTGACGACATTAT |
| q*OsRR8* | CCAGACATGACCGGCTATAACC | AAGCAATTACAACCGGGAGATG |
| q*OsRR9* | GTAACCCCACAAGCGAGAAC | GTATGTCAAAAACCGATCAGACAG |
| q*OsRR10* | CCACTGTTGATTCGGGGA | TGAGTAGAAATTGGGCTGTCC |
| q*ORR1* | GGACTCTGGCTTACTCTC | ACCATAACTGTCAACATTCG |
| q*ORR2* | GGACCATACCAACCTACC | AATCATTATCATCGCCTTCATC |
| q*ORR3* | TCAAGTTCGTATGGAGTTCTGT | TGCTACTGTTACTACTGCTATTGT |
| q*ORR4* | AAGCCAGTGCGTCTTGAG | CAGCGTCATCATCGTTACCA |
| q*ORR5* | GGTGTTGGATACAGACGATT | CAGAATGGCAAGATGGAGAG |
| q*ORR6* | TGAACGCTGTGACCTATT | TCCATACCGCAACTCATT |
| q*OsCKX1* | TCAACAAATCCAAGTGGGATGCGG | TCGCAGAACCTCAGTATCCTCCTGT |
| q*OsCKX2* | GTCAGTGGAGGGGCGGTA | CGTTGGAAATCTGGGGGC |
| q*OsCKX3* | ATGAGCAATCCCTTCACAGCTCCT | TGACTTCCACGACCTGTTCCACAT |
| q*OsCKX4* | GACCGACTACCTCCATCTCACA | GGTTGACATTGCTGACCTGC |
| q*OsCKX5* | AGGGCCTAATCAACAACTGGAGGT | GGTGGAGTCGTCGTAATTCTTGGT |
| q*OsCKX6* | ACAAGTCCAGCTCAATCGGACACT | TCCCGCAAGCCTATCAATGTCGAA |
| q*OsCKX8* | CATCAGTGGCCAAACGTTCAAGCA | AGTTGGAGTGGAATCCTTGCCCTT |
| q*OsCKX9* | GCCTTCCTCCTTATTCCACA | TGGGACCATTGTTACTGTCTTTC |
| q*OsCKX10* | ACTTGAACCAGCTCCGAAAAGA | TGCAGAGAAGAAGGAAGACGAC |
| q*OsCKX11* | TGGCGAGATCTTCTACCTGGTG | CAATGATTGCGTTGTTCTGCGCCA |
| q*D3* | TCCAAACTTGCGGGACATGCAGTT | CCATTGCACAGTGGAGCAATGGCA |
| q*D10* | CTGTACAAGTTCGAGTGGCACC | CCTCGTCCGTCTCCTCGTAC |
| q*D14* | GTGCTGTCGCATGGCTTC | GCAGGTCGTCGACGTAGG |
| q*D17* | GAGGATGGTGGCTATGTTCT | AGACTGGATCTGATGCTTGCT |
| q*D27* | TCTGGGCTAAAGAATGAAAAGGA | AGAGCTTGGGTCACAATCTCG |
| q*D53* | TGGGTTCTGAGTGCATGTTG | GTGTCCTCACAGGCCACTAG |
| Q*Os900* | GGCTTCTCTGCTTGCTGCTTC | ACCAACGGTTGCCTTCCC |
| Q*Os1400* | ACGACGGCGTTCACTCTCTC | TCCGAACCCGTCAATCTCC |
| q*FC1* | TCGTCCACCAATCTTGTGAGCACC | GTTGGCGAACGCCATGATCACGTC |
